# Antioxidative and Antidiabetic Effects of Natural Polyphenols and Isoflavones

**DOI:** 10.3390/molecules21060708

**Published:** 2016-05-30

**Authors:** Aya Umeno, Masanori Horie, Kazutoshi Murotomi, Yoshihiro Nakajima, Yasukazu Yoshida

**Affiliations:** Health Research Institute, National Institute of Advanced Industrial Science and Technology (AIST), 2217-14 Hayashi-cho, Takamatsu, Kagawa 761-0395, Japan; a-umeno@aist.go.jp (A.U.); murotomi@aist.go.jp (K.M.); y-nakajima@aist.go.jp (Y.N.); yoshida-ya@aist.go.jp (Y.Y.)

**Keywords:** diabetes, oxidative stress, oleuropein, hydroxytyrosol, catechin, chlorogenic acids, hesperidin, isoflavone, insulin secretion, glucose tolerance

## Abstract

Many polyphenols that contain more than two phenolic hydroxyl groups are natural antioxidants and can provide health benefits to humans. These polyphenols include, for example, oleuropein, hydroxytyrosol, catechin, chlorogenic acids, hesperidin, nobiletin, and isoflavones. These have been studied widely because of their strong radical-scavenging and antioxidative effects. These effects may contribute to the prevention of diseases, such as diabetes. Insulin secretion, insulin resistance, and homeostasis are important factors in the onset of diabetes, a disease that is associated with dysfunction of pancreatic β-cells. Oxidative stress is thought to contribute to this dysfunction and the effects of antioxidants on the pathogenesis of diabetes have, therefore, been investigated. Here, we summarize the antioxidative effects of polyphenols from the perspective of their radical-scavenging activities as well as their effects on signal transduction pathways. We also describe the preventative effects of polyphenols on diabetes by referring to recent studies including those reported by us. Appropriate analytical approaches for evaluating antioxidants in studies on the prevention of diabetes are comprehensively reviewed.

## 1. Introduction

Insulin resistance is considered to be an important risk factor for the onset of type 2 diabetes. The indirect common cause of insulin resistance is obesity; however, most obese humans adapt to chronic insulin resistance before its onset. For example, humans increase β-cell mass [1] and insulin secretion [2]. β-cell mass is adaptively regulated in response to changes in insulin sensitivity.

Oxidative stress is widely accepted to be involved in the pathogenesis of type 2 diabetes by affecting insulin sensitivity or β-cell mass directly. Interestingly, a study on rodents revealed that gene expression levels of antioxidant enzymes, such as superoxide dismutase (SOD), glutathione peroxidase, and catalase, were much lower in β-cells than in other tissues [3]. Accordingly, oxidative stress could be the major factor in β-cell mass decrease.

Antioxidants have been extensively investigated because of their ability to promote disease prevention and health maintenance by suppressing oxidative stress. Polyphenols are known as potent antioxidants that can contribute to the prevention of type 2 diabetes through their anti-inflammatory, antimicrobial, and immunomodulatory properties. The primary activity of antioxidants is free radical-scavenging. Traditionally, this radical-scavenging effect has been investigated using chemical radical initiators. The stoichiometric number (*n*) of antioxidants that scavenge the number of radicals per one antioxidant molecule, and the effectiveness of radical-scavenging (R_IH_), have been assessed in *in vitro* experiments. Recently, the biological functions of antioxidants have been widely assessed from the perspective of effects on the expression of antioxidant enzymes. It is important to define both properties (*i.e.*, free radical-scavenging activity and effect on enzyme expression) for a complete evaluation of the *in vivo* physiological effects, and biological fate, of an antioxidant. For example, γ-tocopherol is a relatively mild radical-scavenger when compared with α-tocopherol. However, the oxidized product, γ-tocopheryl quinone, reacts readily with thiols to release nuclear factor (erythroid-derived 2)-like-2 (Nrf-2) resulting in the expression of antioxidant enzymes, such as heme oxygenase-1 (HO-1) [4].

In this review article, we describe evaluation methods for assessing radical-scavenging effects quantitatively and for assessing oxidative stress status *in vivo*, especially in the early stage of diabetes onset. In addition, we review recent studies on the role of polyphenols, from a diverse source of foodstuffs, in the prevention of type 2 diabetes.

## 2. Evaluation Methods for Antioxidants

Direct measurements of free radicals are challenging because of their reactivity and short life-spans. Therefore, it is the oxidation products of antioxidants and radicals that are generally measured as a means to assess radical involvement and antioxidant efficacy.

### 2.1. Measurement Methods Using Chemical Reactions

#### 2.1.1. DPPH (1,1-Diphenyl-2-picrylhydrazyl) Radical Scavenging Activity

The absorbance decay of the stable radical 2,2-diphenyl-1-picrylhydrazyl (DPPH) measured spectrophotometrically has been used for evaluation of the efficacy of radical scavengers [5]. The DPPH radical is dark-colored with a maximum absorbance at 517 nm. When the DPPH radical is reduced, it forms a colorless compound. When measuring antioxidant activity with this method, the antioxidant is mixed with the DPPH radical and absorbance is monitored at 520 nm over a given time period. Antioxidant activity is evaluated by comparison with the effects of a known concentration of Trolox^®^ (6-hydroxy-2,5,7,8-tetramethylchroman-2-carboxylic acid). Trolox is a vitamin E mimic and water-soluble antioxidant with well-known kinetic properties. The DPPH radical method can be used in a 96-well plate format with a plate reader. It is, therefore, highly effective for analysis of a large number of samples. However, the method is not suitable if an antioxidant, such as anthocyanin, has the same absorbance as the DPPH radical.

#### 2.1.2. Oxygen Radical Absorbance Activity

The oxygen radical absorbance activity (ORAC) method is one of the main methods for evaluation of the antioxidant activity of food [6]. This method measures the oxidative degradation of the synthetic compound, fluorescein. The peroxyl radical, generated from AAPH (2,2’-azobis (2-methylpropionamidine) dihydrochloride) as the radical initiator, is exposed to fluorescein and the antioxidant sample. The fluorescence intensity of fluorescein is then measured over time. This intensity will decrease depending on the strength of antioxidant activity. In this method, the difference in the area under the curve (AUC) for the antioxidant sample and for a blank is calculated (net AUC). The antioxidant activity is then evaluated by comparison of the net AUC obtained from a known concentration of Trolox^®^. There are two basic ORAC methods: hydrophilic ORAC (H-ORAC) and lipophilic ORAC (L-ORAC). H-ORAC is applied to water-soluble antioxidants, such as polyphenols and ascorbic acid. L-ORAC is applied to hydrophobic antioxidants such as tocopherols. There are a number of variations of the ORAC methodology; we can evaluate a variety of ROS scavengers based on mechanism by changing the kind of radical initiator used. Variations include superoxide anion radical quenching (SORAC) [7], singlet oxygen quenching (SOAC) [8], peroxynitrite quenching (NORAC), and hydroxyl radical quenching (HORAC) [6]. On the other hand, ORAC is not suitable for evaluation of the antioxidant activity of carotenoids. This is because the ORAC method is based on a hydrogen atom transfer mechanism, whereas the antioxidant activity of carotenoids is based on singlet oxygen addition mechanism. Results from the ORAC method and from other *in vitro* antioxidant methods are not always correlated. Additionally, the ORAC method cannot provide information on the effectiveness of radical-scavenging (R_IH_) but it can provide the stoichiometric number of antioxidants (*n*).

#### 2.1.3. Evaluation Method for Radical-Scavenging Property

**Figure 1:**
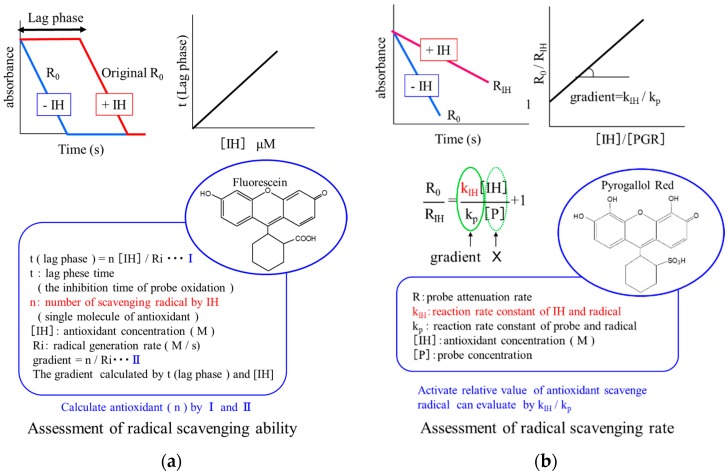
Assessment of (**a**) amount of radicals scavenged by antioxidant (stoichiometric number of antioxidants (*n*)) and (**b**) the effectiveness of radical-scavenging (RIH).

There are numerous methods for evaluating antioxidative activity *in vitro* [9]. For example, (Trolox)-equivalent antioxidant capacity, ferric-reducing antioxidant power, and ORAC are frequently used because of their ease of use and the ready availability of instrumentation. However, many studies have reported inconsistent results using these methods [10,11]. This may be due to the fact that the methods employed measure different actions under different conditions [10,11]. Thus, the development and standardization of a reliable procedure is needed. We have recently proposed a double-assessment method using strong and mild radical scavengers, which provides both the rate and amount of radical-scavenging or the effectiveness of radical-scavenging (Ri) and the stoichiometric number of antioxidants (*n*), respectively (Figure 1) [10]. Briefly, the method involves the following compounds: hydrophilic AAPH, fluorescein, pyrogallolsulfonephthalein (PGR), and water-soluble polyphenols (e.g., oleuropein, hydroxytyrosol, and homovanillic alcohol as the test antioxidants). The assay is started by addition of AAPH to a mixture of PGR (or fluorescein), and the antioxidants in phosphate-buffered saline (PBS). The rates of reaction of fluorescein and PGR with free radicals can be measured by monitoring the decay in absorption at 494 and 540 nm, respectively. The lag phase is obtained graphically by extrapolating the slope of maximum probe (fluorescein) decay to the intersection with the slope of minimum probe decay at the initial stage of the reaction. The rate of PGR consumption is measured from the slope of the decay curve against time at the initial stage. As for other methods, this assay uses Trolox as a reference material. As Trolox is a water-soluble compound, it is an appropriate reference material for this method.

#### 2.1.4. β-Carotene Bleaching Method

This method is based on the color degradation of β-carotene [12]. This involves the reaction of a double bond in β-carotene with a peroxide of auto-oxidized linoleic acid. Absorbance of β-carotene (470 nm) is decreased by reaction with the peroxide. Autoxidation of linoleic acid is promoted by heating an emulsion of linoleic acid, β-carotene, and detergent. The reaction time is less than 1 h and requires little antioxidant sample.

### 2.2. Measurement Methods Based on Enzymatic Reactions (Superoxide Dismutase-Like Activity)

Superoxide dismutase (SOD) is one of the major antioxidant enzymes. SOD catalyzes the following reaction:

2O^・^_2_^-^ + 2H → H_2_O_2_ + O_2_ (1)

In other words, SOD degrades the superoxide radical to hydrogen peroxide and oxygen. Antioxidant activity can be evaluated as SOD-like activity. Available methods for assessing SOD-like activity include that based on the luminol reaction [13]. When oxidized by H_2_O_2_, luminol becomes luminous at 460 nm. The chemiluminescence intensity of luminol upon reaction with superoxide radical can be measured.

Another method for assessing SOD-like activity is the water-soluble tetrazolium (WST-1) assay. WST-1 is formed by reduction of formazan. The absorbance of water-soluble formazan is measured at 450 nm [14]. And MTS assay and XTT assay can be applied for assessing SOD-like activity [15].

Electron spin resonance (ESR) methods can be used: here, stable radical compounds formed with 5,5-dimethyl-1-pyrroline-1-oxide (DMPO) or α-phenyl-*N*-tert-butylnitrone (PBN) were measured by ESR. These methods are often referred to as “spin-traps”.

### 2.3. Evaluation Method for the Early Stage of Oxidative Stress in Vivo

Lipid peroxidation products have received considerable attention as early stage biomarkers because lipids are highly susceptible to oxidation *in vivo*. Traditionally, thiobarbituric reactive substances (TBARS), malondialdehyde, short chain alkanes, and lipid hydroperoxides have been assessed as lipid peroxidation products.

The first attack of radicals formed *in vivo* is directed against lipids. The susceptibility of lipids towards radicals is associated with high levels of polyunsaturation in fatty acid chains. The choice of detection method for the very small amounts of oxidized products typically generated is very important. The most sensitive method for the quantitative analysis is liquid chromatography-mass spectrometry (LC-MS). It is also important to determine what type of oxidized lipid product to measure. In our studies, we have targeted the oxidation products of linoleates. These are much more abundant *in vivo* than other fatty acids. They also contain bis-allylic hydrogens; so oxidation proceeds by a more straightforward mechanism that yields much simpler products than oxidation of arachidonates, or of more highly unsaturated fatty acids, such as docosahexaenoates.

Hydroperoxyoctadecadienoic acids (HPODEs) formed by a radical-mediated oxidation mechanism comprise 4 isomers: 13-hydroperoxy-9(*Z*), 11(*E*)-octadecadienoic acid (13-(*Z*,*E*)-HPODE), 13-(*E*,*E*)-HPODE, 9-(*E*,*Z*)-HPODE, and 9-(*E*,*E*)-HPODE. Very little 11-HPODE is formed under normal conditions because the pentadienyl radical that is formed by the abstraction of hydrogen at carbon 11 rapidly rearranges to form stable conjugated diene radicals. The isomers, 9- and 13-(*Z*,*E*)-HPODE, are also formed by enzymatic oxidation via lipoxygenase as enantio-, regio-, and stereo-specific products. Thus, 9- and 13-(*E*,*E*)-HPODE are specific products of radical-mediated oxidation.

On the other hand, singlet oxygen oxidizes linoleic acid by non-radical oxidation to form 13-(*Z*,*E*)-HPODE, 10-hydroperoxy-8(*E*), 12(*Z*)-octadecadienoic acid (10-(*E*,*Z*)-HPODE), 12-hydroperoxy-9(*Z*), 13(*E*)-octadecadienoic acid (12-(*Z*,*E*)-HPODE), and 9-(*E*,*Z*)-HPODE. In this case, 10- and 12-(*Z*,*E*)-HPODEs are specific oxidation products from reactions involving singlet oxygen.

Cholesterol oxidation products, commonly referred to as oxysterols, have received increasing attention as diagnostic biomarkers of oxidative stress, as intermediates in bile acid biosynthesis, and as messengers for cell signaling and cholesterol transport [16]. Cholesterol is oxidized by both enzymatic and non-enzymatic mechanisms. The free radical-mediated oxidation of cholesterol yields 7α- and 7β-hydroperoxycholesterol (7α- and 7β-OOHCh), 7α- and 7β-OHCh, 5α,6α- and 5β,6β-epoxycholesterol, and 7-ketocholesterol (7-KCh) as major products [16]. The conversion of 7-KCh into 7β-OHCh *in vivo* has been previously reported [17]. The oxidation of 7-OHCh by either 7α-hydroxycholesterol dehydrogenase [18] or by non-enzymatic autoxidation yields 7-KCh [16]. 7β-OHCh may be regarded as a marker of free radical-mediated oxidation.

The oxidation of cholesterol by singlet oxygen gives 5α- and 5β-OOHCh, and then 5-OHCh. Oxysterols are present *in vivo* in different forms, namely the esterified, sulfated, and conjugated forms, as well as free oxysterols [19]. We have recently presented LC-MS/MS and GC-MS methods for determining oxidation products of linoleates and cholesterol, respectively [20,21,22,23,24]. Briefly, physiological samples are mixed with saline solution and methanol containing the internal standards 8-iso-PGF2α-d4, 13-HODE-d4, and 7β-OHCh-d7. Butylated hydroxytoluene is then added to the samples. This is followed by reduction of hydroperoxides using excess triphenylphosphine followed by saponification using KOH in methanol under nitrogen. The mixture is then acidified with acetic acid in water, and extracted with chloroform and ethyl acetate. The extracted sample is divided into two equal portions. The first portion is subjected to LC-MS/MS for analysis of isoprostanes and HODEs. The second portion is treated with a silylating agent and injected into a GM equipped with a quadrupole mass spectrometer for cholesterol and linoleates analysis.

## 3. Antioxidative and Antidiabetic Effects of Polyphenols

### 3.1. Olive Leaf: Oleuropein and Hydroxytyrosol

For several thousand years, the Mediterranean diet has included an abundant amount of olive oil. Several epidemiological studies suggest that the Mediterranean diet is effective for prevention of cardiovascular diseases and diabetes [25,26]. Olive oil has shown positive effects in diabetes-related early events, both in animals and humans [27,28,29]. Although olive oil reduces the risk of cardiovascular diseases, contribution of monounsaturated fatty acids and oleic acid to this beneficial effect was minimal [30]. Olive oil includes high amounts of phenols and polyphenols [31,32]. These observations suggest the possibility that phenols and polyphenols are important for this disease prevention.

The major phenol of olive fruits and leaves is oleuropein (OP). It is present in leaves at greater levels than in fruits [33]. OP olive is a heterosidic ester comprised of hydroxytyrosol (HT) and β-glucosylated elenolic acid [34] and it exhibits antioxidant and free radical-scavenging activities. OP is metabolized to HT by hydrolysis, and HT is known to have beneficial effects. However, numerous reports have been published on the direct beneficial properties of OP (rather than the HT product). These include lowering of blood pressure [35], inhibition of platelet aggregation [36], cardio-protection [37], and anticancer activity [38]. Mechanisms of OP disease prevention include decreased expression of genes involved in adipogenesis, e.g., PPARγ, lipoprotein lipase, and fatty acid-binding protein 4, and reduced fat accumulation [39]. OP also scavenges superoxide anions and hydroxyl radicals, and inhibits the respiratory burst of neutrophils and hypochlorous acid-derived radicals [40]. As mentioned earlier, HT is released from OP under acidic environments, such as the stomach [41]. The effects of HT against disease *in vivo* has been described frequently [42,43,44,45,46]. HT inhibits hydrogen peroxide-induced kidney cell injury by interacting with MAP kinase and PI3 kinase [47]. It inhibits lipid peroxidation in intestinal Caco-2 cells by scavenging peroxyl radicals [48] and induces heme oxygenase 1 gene expression in human keratinocytes [49].

Earlier *in vitro* studies, have revealed the following: OP and HT react with the stable radical 2,2-diphenyl-1-picrylhydrazyl (DPPH) and hydroxyl radical [50]. OP and HT are also potent scavengers of peroxynitrite and superoxide anion radicals, but not of hypochlorous acid or hydrogen peroxide [51]. However, the reactivity of OP and HT toward free radicals has not been systematically evaluated. Neither has it been clarified whether an OP-rich diet suppresses the onset of diabetes. Our recent studies have provided findings to address these issues. OP and HT act as scavengers of oxygen radicals. However, based on assessment with our PGR method, the reactivity of OP and HT is mild when compared with Trolox. The fluorescein method revealed that an OP or HT molecule is able to scavenge more than two oxygen radicals. Thus, it is suggested that these compounds may play a critical role in the inhibition of lipid peroxidation. Furthermore, their activity might be increased substantially by the presence of nucleophiles *in vivo* [52]. An OP-rich diet (content of OP was greater than 35%, *w*/*w*) cause a mild reduction of oxidative stress, as assessed by total levels of HODE, in Tsumura Suzuki Obese Diabetes (TSOD) mice, and attenuated anxiety-like behavioral abnormality in aged TSOD mice [53].

### 3.2. Tea Polyphenols: Catechins and Theaflavins

The major polyphenols in tea (*Camellia sinensis*) are catechins. Catechin (flavan-3-ol) is a member of the flavonoids. Catechin possesses two benzene rings (termed the A- and B-rings) and a dihydropyran heterocycle (the C-ring). Catechin has hydroxyl groups at C-5 and C-7 in the A-ring, at C-3’ and C-4’ in the B-ring, and at C-3 in C-ring. The catechins found in tea include mainly epicatechin (EC), epigallocatechin (EGC) which is a dihydroxy analog of EC, epicatechin gallate (ECg), and epigallocatechin gallate (EGCg). These catechins have antioxidant activities [54].

There are three types of tea based on fermentation status; unfermented tea (green tea), fermented tea (enzymatic fermentation), such as black tea and oolong tea, and post-fermented tea (microbial fermentation) such as Pu-erh tea, miang, and Kuro-cha. Generally, the catechin content in unfermented tea (green tea) is approximately 15%. This catechin content decreases during fermentation. The catechin content of oolong tea and black tea is approximately 4%–8% [55]. On the other hand, other polyphenolics become more predominant in these teas. In oolong tea, levels of procyanidin and theasinensins are increased and, in black tea, theaflavin content is increased. These compounds are formed by condensation of two catechin moieties and are differentiated by the different condensation routes.

Of these teas, green tea has the largest radical scavenging ability against hydroxyl radical generated by the Fenton reaction. One study showed that 86% of radicals were removed by green tea, whereas the radical scavenging abilities of oolong tea and black tea were approximately 50% [56]. Superoxide anion scavenging ability is also greatest in green tea. Green tea could scavenge 100% of superoxide anions, whereas the superoxide anion scavenging abilities of oolong tea and black tea were approximately 60% [56].

Catechins have other physiological activities besides *in vitro* antioxidative activity. It has been reported that catechins confer antioxidant properties to cells through activation of the Nrf2 pathway [57,58]. EGCg conferred a cytoprotection effect by induction of heme oxygenase-1 (HO-1), a major antioxidative protein, via Nrf2 activation in rat neuronal cells [59]. Catechins also enhanced adipocyte differentiation by activation of PPARγ [60]. Additionally, intake of catechin-rich green tea slightly inhibited postprandial elevation of blood glucose levels and oxidative products in postmenopausal women [61]. These effect suggest that intake of green tea containing catechins could reduce the risk of type 2 diabetes.

As mentioned above enzymatic fermented teas, such as oolong tea and black tea, have a different polyphenol composition from that of green tea. Enzymatic fermented tea contains theaflavin as a major polyphenol. Catechin is converted to theaflavin and thearubigin through oxidative polymerization catalyzed by polyphenol oxidase (PPO) during the fermentation process [62,63]. Theaflavin is reddish in color, so the color of black tea depends on the amount of theaflavin generated during fermentation. Theaflavin has been shown to have a cholesterol-lowering effect [64]; intake of capsules containing theaflavin-enriched green tea extract for 12 weeks decreased levels of total cholesterol and LDL cholesterol. Theaflavins and theasinensin also demonstrated antihyperglycemic and hypotriacylglycerolemic effects in rat [64]. Based on animal and cell experiments, the inhibitory effect of postprandial hyperglycemia induced by black tea is caused by reduced polysaccharide degradation and intestinal absorption due to inhibition of α-glucosidase activity in the small intestine [65]. Although the association between the α-glucosidase inhibitory effect and polyphenols in tea is unclear, intake of fermented tea shows a diabetes preventative effect.

Finally, post-fermentation tea is produced through fermentation with microorganisms, such as fungi or lactic acid bacteria. Generally, the catechin content in post-fermentation tea is lower than that in green tea. However, post-fermented tea has the same level of antioxidative capacity as green tea. Although there is a possibility that oxidative polymerization of catechins is effected by microorganisms during the fermentation process, the polyphenolic composition of post-fermentation tea is yet to be fully characterized.

### 3.3. Cocoa Polyphenols: Catechins

The major polyphenolic components in cocoa are catechin, EC, and procyanidin B2. These components have strong antioxidative activity [66]. The physiological properties of cocoa polyphenols have been reported. A polyphenol-rich cocoa extract was shown to have antioxidative activity [54], as well as α-amylase and α-glycosidase inhibitory activities [67]. Aqueous extract of cocoa bean powder which including 17.9 ± 0.96 (mgGAE/100 g) polyphenols showed DPPH radical, OH radical, and NO radical scavenging ability, and the aqueous extract prevent lipid peroxidation of rat pancreas induced by sodium nitrite and Fe^2+^. The aqueous extract also inhibited α-amylase and α-glycosidase activity [68]. Additionally, it was reported that polyphenol-rich chocolate improved type 2 diabetes [69]. However, chocolate also contains sugars and fatty ingredients. It would, therefore, be necessary to establish a balance between these components and polyphenols to ensure an antidiabetic effect from chocolate.

### 3.4. Coffee Polyphenol: Chlorogenic Acid

The hydroxyl radical scavenging ability of instant coffee, as measured by the ESR spin trapping method, was shown to be 20 times greater than that of blueberry [70]. The major polyphenol in coffee is chlorogenic acid. It is also present in dicot plant vegetables such as cowpea and burdock root [58]. Chlorogenic acid has radical trapping and singlet oxygen removal capacity. It can prevent LDL oxidization and oxidative injury to nucleic acids [71,72,73,74,75]. Chlorogenic acid has also been reported to have effects associated with the prevention of diabetes. It inhibited α-glycosidase activity and inhibited postprandial elevation of blood glucose levels in sucrose- and maltose-treated rats [63]. It has been reported that chlorogenic acid and its isomers which were extracted from coffee bean by supercritical extraction (CO_2_, 70 °C, 45 MPa) and alcohol extraction (60% ethanol, 50 °C, 60 min) contributed 60%–85% of the inhibition effect of coffee extract on carbohydrate degradation enzyme, maltase, sucrose, and α-amylase activity [76]. Chlorogenic acid, therefore, contributes to inhibition of postprandial elevation of blood glucose levels via inhibition of carbohydrate degradation. Intake of chlorogenic acid-rich coffee extract suppressed fat utilization in humans [77]. Intake of coffee polyphenols drove secretion of glucagon-like peptide 1 (GLP-1), which has been shown to exhibit an antidiabetic effect, resulting in reduction of blood glucose levels [78]. Moreover, continuous drinking of coffee has been shown to reduce levels of visceral fat [79]. Chlorogenic acid reduced blood LDL levels in hypercholesterolemic rats [80]. Administration of chlorogenic acid to golden hamsters enhanced expression of PPARα in liver and total cholesterol, LDL, HDL, glucose, and insulin levels in blood were lower than in the placebo group [81]. These results suggested that chlorogenic acid affected lipid metabolism through activation of PPARα in liver. Overall, chlorogenic acid has a glucose absorption inhibitory effect and a fat combustion effect. These properties are valuable for the prevention of type 2 diabetes.

### 3.5. Citrus Polyphenols: Hesperidin and Nobiletin

Citrus fruits contain polyphenols that have antioxidative and antidiabetic activity. Polyphenols in citrus are mainly contained in the peel. One of the major polyphenols of citrus is hesperidin. Hesperidin is a flavonoid found in a variety of Citrus species including *C. unshiu*, *C. hassaku*, and *C. aurantium*. The aglycone of hesperidin is herperetin. Both hesperidin and herperetin have radical trapping ability and antioxidative activity [82].

The major physiological property of hesperidin is its anti-inflammatory activity: hesperidin decreased gene expression of cyclooxygenase-2 (COX-2) [82,83]. There are also reports on the antidiabetic effect of hesperidin [84,85]. Feeding of 1% hesperidin prevented elevation of blood glucose level and serum insulin level in Goto-Kaizaki (GK) rat. And the mRNA level of PPARα and PPARγ in the hesperidin fed GK rat was significantly higher than control animals [84]. Plasma insulin level and glucokinase were elevated in hesperidin-supplemented fed C57BL/KsJ-db/db mice [85] Administration of hesperidin to a diabetic rat model rat reduced blood levels of HbA1c, glucose, CES, total cholesterol, and triglycerides l [86]. In addition, blood LDL levels were decreased and HDL levels were increased in hesperidin-treated rats [87]. Hesperidin activated PPARγ in diabetic rat and caused a reduction in blood lipid peroxide levels [88]. Glucosyl-hesperidin, which is formed by intestinal α-glycosidase, also has radical trapping capacity [89]. It has been shown to reduce blood triglyceride levels and improve abnormal LDL metabolism [90,91,92].

Another important polyphenol in citrus is nobiletin. As with hesperidin, nobiletin is a flavonoid with antioxidative, anti-inflammatory, and antidiabetic activity [93]. When nobiletin was administered to high-calorie diet fed mice, inhibition of blood sugar and insulin elevation, and decrease of leptin concentration and adipocyte diameter were observed. However, nobiletin has not been shown to activate PPAR and thus these effects may be PPAR-independent [94]. Sudachitin is a polyphenol found in *C. sudachi*. Sudachitin has been reported to have antioxidative activity [95]. It also prevented blood sugar and insulin elevation in high-fat diet fed mice [96]. Eriocitrin (flavanone-7-*O*-glycoside) is a found in lemon (*C. limon*) and lime (*C. aurantifolia*). It has stronger antioxidative activity than hesperidin [97] and has been reported to suppress lipid peroxidation in liver [98].

### 3.6. Soybean: Isoflavones

Isoflavones are a type of flavonoid found in leguminous plants such as soybean (*Glycine max*) and kudzu (*Pueraria lobata*). Typical isoflavones include genistein, daidzein, and puerarin (the 8-*C*-glucoside of daidzein). Isoflavones are known as phytoestrogens and have estrogen-like activity when administered to mammals. On the other hand, recent investigations suggest that the functional mechanisms of isoflavones and estrogen are different [99]. Genistein acts as an antioxidant and can reduce free radical related tissue injury [100]. According to evaluation of radical scavenging activity of genistein by ESR, the multiple ROS scavenging rate of genistein is similar to that of glutathione and it has singlet oxygen removal capacity [73]. Genistein activates PPARγ and enhances expression of superoxide dismutase (SOD) and catalase via Nrf2 activation in EA.hy926 cells [101]. The genistein glycoside, genistein-7-*O*-gentiobioside, which is found in groundnut (*Apios americana*) has been shown to enhance HO-1 expression and exert antioxidative activity in human breast cancer MCF-7 cells. However, genistein-7-*O*-gentiobioside has very little antioxidative activity [102].

Isoflavones are also associated with an antidiabetic effect. For example, isoflavones reduced diabetes risk in females. According to the epidemiological study in Korea, plasma concentration of genistein correlated to decrease of risk of type 2 diabetes in women [103]. Interestingly, the antidiabetic effect of isoflavones was not observed in males. Activation of the antioxidative system of cells is important for the biological effects of isoflavones. There is also an association of the antidiabetic effect of isoflavone and their estrogen-like activity. The mechanisms by which isoflavones exert their physiological effects appear to be different from those of the other polyphenols discussed in this section.

### 3.7. General Overview

**Figure 2:**
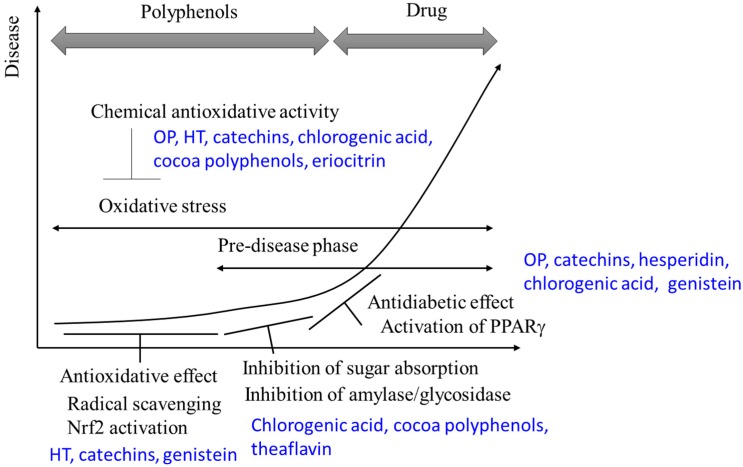
The preventative effect of polyphenol at each stage of diabetes.

Most polyphenols inhibit amylase and glycosidase activity and, thus, inhibit glucose absorption in the intestine. In addition, polyphenols activate PPARγ and promote adiponectin production, thus subsequently improving insulin resistance. The antioxidative properties and antidiabetic efficacy of polyphenols are independent of each other and their interrelationship is still unclear. Nonetheless, in pre-symptomatic states, polyphenols contribute to the prevention of the type 2 diabetes through antioxidative activity. In the early phase of type 2 diabetes, polyphenols alleviate symptoms through PPARγ activation and inhibition of glucose absorption (Figure 2). Understanding the interaction of the antioxidant activities and antidiabetic effects of polyphenols will need to be clarified in the future. This will require effective means to measure the chemical antioxidative activity of polyphenols *in vivo*, which currently remain a significant challenge.

## 4. Conclusions and Outlook

During pre-symptomatic states, borderline diabetes, or onset of type 2 diabetes, oxidative stress in the body is increased. In the early stages of type 2 diabetes, blood levels of the lipid oxidation product, HODE, are increased. An increase in HODE levels indicates oxidative stress and, in particular, the involvement of radicals and singlet oxygen. Inhibition of oxidative stress may, therefore, be effective for the prevention of type 2 diabetes in the early stages, including pre-symptomatic states. When type 2 diabetes is in onset, chronic elevation of blood sugar level and insulin resistance is observed. Thus, everyday intake of polyphenols could inhibit increases in oxidative stress and, thus, reduce the risk of developing type 2 diabetes.

Polyphenols show strong antioxidant activity in *in vitro*. The antioxidant activity of polyphenols results in the removal of reactive oxygen and a decrease in oxidative stress. However, direct measurement of the antioxidant activity of polyphenols is difficult in *in vivo*. It is also unclear whether the antioxidant activity of polyphenols observed *in vitro* will correlate with efficacy *in vivo*.

Polyphenols can induce antioxidative effects in cultured cells via activation of the Nrf2 pathway and subsequent expression of antioxidative proteins, such as HO-1. Intake of polyphenols can, therefore, decrease oxidative stress through either intrinsic chemical antioxidant property or induction of antioxidative properties within cells.

Generally, many kinds of polyphenols show both the antioxidant activity and the antidiabetic activity. However, when we take polyphenols from a food, its effect may not be same as the effect as the chemicals. Antioxidative and antidiabetic activity may change by processing methods, such as heating. Concentration of polyphenols in peel, pulp, and seed of fruit is different. Generally, we eat only juice sacks of unshu mikan (*C. unshiu*). On the other hand, we use the peel of oranges, yuzu (*C. junos*), and lemon to make marmalade. For example, extra virgin olive oil (EVOO) includes polyphenol more than refined olive oil, and the antioxidant activity of EVOO is also strong [31,32]. Total antioxidant activity attributable to polyphenols of EVOO was 16–57 times stronger than that of refined olive oil [32]. How to eat a food including polyphenols is important to their expected antioxidative and antidiabetic effects.

## Abbreviations

The following abbreviations are used in this manuscript:

AAPH: 2,2’-azobis(2-amidinopropane) dihydrochloride
DPPH: 2,2-diphenyl-1-picrylhydrazyl
GSH: glutathione
HA: homovanillic alcohol
HT: hydroxytyrosol
OP: oleuropein
PGR: pyrogallol red
Trolox: 2-carboxy-2,5,7,8-tetramethyl-6-chromanol

## References

[1] Butler A.E., Janson J., Bonner-Weir S., Ritzel R., Rizza R.A., Butler P.C. (2003). Beta-cell deficit and increased beta-cell apoptosis in humans with type 2 diabetes. Diabetes.

[2] Polonsky K.S. (2000). Dynamics of insulin secretion in obesity and diabetes. Int. J. Obes. Relat. Metab. Disord..

[3] Lenzen S., Drinkgern J., Tiedge M. (1996). Low antioxidant enzyme gene expression in pancreatic islets compared with various other mouse tissues. Free Radic. Biol. Med..

[4] Ogawa Y., Saito Y., Nishio K., Yoshida Y., Ashida H., Niki E. (2008). Gamma-tocopheryl quinone, not alpha-tocopheryl quinone, induces adaptive response through up-regulation of cellular glutathione and cysteine availability via activation of ATF4. Free Radic. Res..

[5] Foti M.C. (2015). Use and Abuse of the DPPH (•) Radical. J. Agric. Food Chem..

[6] Amorati R., Valgimigli L. (2015). Advantages and limitations of common testing methods for antioxidants. Free Radic. Res..

[7] Yang J., Ou B., Wise M.L., Chu Y. (2014). *In vitro* total antioxidant capacity and anti-inflammatory activity of three common oat-derived avenanthramides. Food Chem..

[8] Takahashi S., Iwasaki-Kino Y., Aizawa K., Terao J., Mukai K. (2016). Development of Singlet Oxygen Absorption Capacity (SOAC) Assay Method Using a Microplate Reader. J. AOAC Int..

[9] Niki E. (2010). Assessment of antioxidant capacity *in vitro* and *in vivo*. Free Radic. Biol. Med..

[10] Takashima M., Horie M., Shichiri M., Hagihara Y., Yoshida Y., Niki E. (2012). Assessment of antioxidant capacity for scavenging free radicals *in vitro*: A rational basis and practical application. Free Radic. Biol. Med..

[11] Takashima M., Shichiri M., Hagihara Y., Yoshida Y., Niki E. (2012). Reactivity toward oxygen radicals and antioxidant action of thiol compounds. Biofactors.

[12] Miller H.E. (1971). A simplified method for the evaluation of antioxidants. J. Am. Oil Chem. Soc..

[13] Lissi E., Pascual C., del Castillo M.D. (1994). On the use of the quenching of luminol luminescence to evaluate SOD activity. Free Radic. Biol. Med..

[14] Peskin A.V., Winterbourn C.C. (2000). A microtiter plate assay for superoxide dismutase using a water-soluble tetrazolium salt (WST-1). Clin. Chim. Acta.

[15] Sutherland M.W., Learmonth B.A. (1997). The tetrazolium dyes MTS and XTT provide new quantitative assays for superoxide and superoxide dismutase. Free Radic. Res..

[16] Diczfalusy U. (2004). Analysis of cholesterol oxidation products in biological samples. J. AOAC Int..

[17] Erickson S.K., Cooper A.D., Matsui S.M., Gould R.G. (1977). 7-Ketocholesterol. Its effects on hepatic cholesterogenesis and its hepatic metabolism *in vivo* and *in vitro*. J. Biol. Chem..

[18] Song W., Pierce W.M., Saeki Y., Redinger R.N., Prough R.A. (1996). Endogenous 7-oxocholesterol is an enzymatic product: Characterization of 7α--hydroxycholesterol dehydrogenase activity of hamster liver microsomes. Arch. Biochem. Biophys..

[19] Brown A.J., Jessup W. (1999). Oxysterols and atherosclerosis. Atherosclerosis.

[20] Yoshida Y., Umeno A., Shichiri M. (2013). Lipid peroxidation biomarkers for evaluating oxidative stress and assessing antioxidant capacity *in vivo*. J. Clin. Biochem. Nutr..

[21] Yoshida Y., Yoshikawa A., Kinumi T., Ogawa Y., Saito Y., Ohara K., Yamamoto H., Imai Y., Niki E. (2009). Hydroxyoctadecadienoic acid and oxidatively modified peroxiredoxins in the blood of Alzheimer’s disease patients and their potential as biomarkers. Neurobiol. Aging.

[22] Umeno A., Shichiri M., Ishida N., Hashimoto Y., Abe K., Kataoka M., Yoshino K., Hagihara Y., Aki N., Funaki M. (2013). Singlet Oxygen Induced Products of Linoleates, 10-and 12-(*Z*,*E*)-Hydroxyoctadecadienoic Acids (HODE), Can Be Potential Biomarkers for Early Detection of Type 2 Diabetes. PLoS ONE.

[23] Umeno A., Yoshino K., Hashimoto Y., Shichiri M., Kataoka M., Yoshida Y. (2015). Multi-biomarkers for early detection of type 2 diabetes, including 10- and 12-(*Z*,*E*)-hydroxyoctadecadienoic acids, insulin, leptin, and adiponectin. PLoS ONE.

[24] Yoshida Y., Umeno A., Akazawa Y., Shichiri M., Murotomi K., Horie M. (2015). Chemistry of lipid peroxidation products and their use as biomarkers in early detection of diseases. J. Oleo Sci..

[25] Soriguer F., Rojo-Martínez G., Goday A., Bosch-Comas A., Bordiú E., Caballero-Díaz F., Calle-Pascual A., Carmena R., Casamitjana R., Castaño L. (2013). Olive oil has a beneficial effect on impaired glucose regulation and other cardiometabolic risk factors. Di@bet.es study. Eur. J. Clin. Nutr..

[26] Sleiman D., Al-Badri M.R., Azar S.T. (2015). Effect of mediterranean diet in diabetes control and cardiovascular risk modification: A systematic review. Front. Public Health.

[27] Guasch-Ferré M., Hruby A., Salas-Salvadó J., Martínez-González M.A., Sun Q., Willett W.C., Hu F.B. (2015). Olive oil consumption and risk of type 2 diabetes in US women. Am. J. Clin. Nutr..

[28] Roche E., Ramírez-Tortosa C.L., Arribas M.I., Ochoa J.J., Sirvent-Belando J.E., Battino M., Ramírez-Tortosa M.C., González-Alonso A., Pérez-López M.P., Quiles J.L. (2014). Comparative analysis of pancreatic changes in aged rats fed life long with sunflower, fish, or olive oils. J. Gerontol. A Biol. Sci. Med. Sci..

[29] Martínez-González M.A., Salas-Salvadó J., Estruch R., Corella D., Fitó M., Ros E. (2015). Benefits of the Mediterranean Diet: Insights from the PREDIMED Study. Prog. Cardiovasc. Dis..

[30] Schwingshackl L., Hoffmann G. (2014). Monounsaturated fatty acids, olive oil and health status: A systematic review and meta-analysis of cohort studies. Lipids Health Dis..

[31] Medina I., Satué-Gracia M.T., German J.B., Frankel E.N. (1999). Comparison of natural polyphenol antioxidants from extra virgin olive oil with synthetic antioxidants in tuna lipids during thermal oxidation. J. Agric. Food Chem..

[32] Pellegrini N., Visioli F., Buratti S., Brighenti F. (2001). Direct analysis of total antioxidant activity of olive oil and studies on the influence of heating. J. Agric. Food Chem..

[33] Zoidou E., Melliou E., Gikas E., Tsarbopoulos A., Magiatis P., Skaltsounis A.L. (2010). Identification of Throuba Thassos, a traditional Greek table olive variety, as a nutritional rich source of oleuropein. J. Agric. Food Chem..

[34] Ortega-García F., Blanco S., Peinado M.A., Peragón J. (2008). Polyphenol oxidase and its relationship with oleuropein concentration in fruits and leaves of olive (*Olea europaea*) cv. ‘Picual’ trees during fruit ripening. Tree Physiol..

[35] Susalit E., Agus N., Effendi I., Tjandrawinata R.R., Nofiarny D., Perrinjaquet-Moccetti T., Verbruggen M. (2011). Olive (*Olea europaea*) leaf extract effective in patients with stage-1 hypertension: Comparison with Captopril. Phytomedicine.

[36] Petroni M.L., Jazrawi R.P., Grundy A., Lanzin I.A., Pigozzi M.G., Biasio A., Heaton K.W., Virjee J., Northfield T.C. (1995). Prospective, multicenter study on value of computerized tomography (CT) in gallstone disease in predicting response to bile acid therapy. Dig. Dis. Sci..

[37] Misra A., Singhal N., Khurana L. (2010). Obesity, the metabolic syndrome, and type 2 diabetes in developing countries: Role of dietary fats and oils. J. Am. Coll. Nutr..

[38] Hamdi H.K., Castellon R. (2005). Oleuropein, a non-toxic olive iridoid, is an anti-tumor agent and cytoskeleton disruptor. Biochem. Biophys. Res. Commun..

[39] Santiago-Mora R., Casado-Díaz A., de Castro M.D., Quesada-Gómez J.M. (2011). Oleuropein enhances osteoblastogenesis and inhibits adipogenesis: The effect on differentiation in stem cells derived from bone marrow. Osteoporos. Int..

[40] Efentakis P., Iliodromitis E.K., Mikros E., Papachristodoulou A., Dagres N., Skaltsounis A.L., Andreadou I. (2015). Effects of the olive tree leaf constituents on myocardial oxidative damage and atherosclerosis. Planta Med..

[41] Corona G., Tzounis X., Assunta Dessì M., Deiana M., Debnam E., Visioli F., Spencer J.P. (2006). The fate of olive oil polyphenols in the gastrointestinal tract: Implications of gastric and colonic microflora-dependent biotransformation. Free Radic. Res..

[42] Granados-Principal S., Quiles J.L., Ramirez-Tortosa C., Camacho-Corencia P., Sanchez-Rovira P., Vera-Ramirez L., Ramirez-Tortosa M.C. (2011). Hydroxytyrosol inhibits growth and cell proliferation and promotes high expression of sfrp4 in rat mammary tumours. Mol. Nutr. Food Res..

[43] Mateos R., Martínez-López S., Baeza Arévalo G., Amigo-Benavent M., Sarriá B., Bravo-Clemente L. (2016). Hydroxytyrosol in functional hydroxytyrosol-enriched biscuits is highly bioavailable and decreases oxidised low density lipoprotein levels in humans. Food Chem..

[44] Granados-Principal S., El-Azem N., Pamplona R., Ramirez-Tortosa C., Pulido-Moran M., Vera-Ramirez L., Quiles J.L., Sanchez-Rovira P., Naudí A., Portero-Otin M. (2014). Hydroxytyrosol ameliorates oxidative stress and mitochondrial dysfunction in doxorubicin-induced cardiotoxicity in rats with breast cancer. Biochem. Pharmacol..

[45] Zheng A., Li H., Cao K., Xu J., Zou X., Li Y., Chen C., Liu J., Feng Z. (2015). Maternal hydroxytyrosol administration improves neurogenesis and cognitive function in prenatally stressed offspring. J. Nutr. Biochem..

[46] Bullon P., Quiles J.L., Morillo J.M., Rubini C., Goteri G., Granados-Principal S., Battino M., Ramirez-Tortosa M. (2009). Gingival vascular damage in atherosclerotic rabbits: Hydroxytyrosol and squalene benefits. Food Chem. Toxicol..

[47] Incani A., Deiana M., Corona G., Vafeiadou K., Vauzour D., Dessì M.A., Spencer J.P. (2010). Involvement of ERK, Akt and JNK signalling in H2O2-induced cell injury and protection by hydroxytyrosol and its metabolite homovanillic alcohol. Mol. Nutr. Food Res..

[48] Deiana M., Corona G., Incani A., Loru D., Rosa A., Atzeri A., Paola Melis M., Assunta Dessì M. (2010). Protective effect of simple phenols from extravirgin olive oil against lipid peroxidation in intestinal Caco-2 cells. Food Chem. Toxicol..

[49] Sgarbossa A., Dal Bosco M., Pressi G., Cuzzocrea S., Dal Toso R., Menegazz I.M. (2012). Phenylpropanoid glycosides from plant cell cultures induce heme oxygenase 1 gene expression in a human keratinocyte cell line by affecting the balance of NRF2 and BACH1 transcription factors. Chem. Biol. Interact..

[50] Valavanidis A., Nisiotou C., Papageorgiou Y., Kremli I., Satravelas N., Zinieris N., Zygalaki H. (2004). Comparison of the radical scavenging potential of polar and lipidic fractions of olive oil and other vegetable oils under normal conditions and after thermal treatment. J. Agric. Food Chem..

[51] Rietjens S.J., Bast A., Haenen G.R. (2007). New insights into controversies on the antioxidant potential of the olive oil antioxidant hydroxytyrosol. J. Agric. Food Chem..

[52] Umeno A., Takashima M., Murotomi K., Nakajima Y., Koike T., Matsuo T., Yoshida Y. (2015). Radical-scavenging activity and antioxidative effects of olive leaf components oleuropein and hydroxytyrosol in comparison with homovanillic alcohol. J. Oleo Sci..

[53] Murotomi K., Umeno A., Yasunaga M., Shichiri M., Ishida N., Koike T., Matsuo T., Abe H., Yoshida Y., Nakajima Y. (2015). Oleuropein-rich diet attenuates hyperglycemia and impaired glucose tolerance in type 2 diabetes model mouse. J. Agric. Food Chem..

[54] Matsuzaki T., Hara Y. (1985). Antioxidative activity of tea leaf catechins. Nippon Nogeikagaku Kaishi.

[55] Sano M., Tabata M., Suzuki M., Degawa M., Miyase T., Maeda-Yamamoto M. (2001). Simultaneous determination of twelve tea catechins by high-performance liquid chromatography with electrochemical detection. Analyst.

[56] Nishizawa C., Nguyen V.C. (2001). The Comparison between Coffee and Teas on Desmutagenicity, Radical Scavenging Activity and Antioxidative Activity. Nippon Shokuhin Kagaku Kogaku Kaishi.

[57] Pullikotil P., Chen H., Muniyappa R., Greenberg C.C., Yang S., Reiter C.E., Lee J.W., Chung J.H., Quon M.J. (2012). Epigallocatechin gallate induces expression of heme oxygenase-1 in endothelial cells via p38 MAPK and Nrf-2 that suppresses proinflammatory actions of TNF-α. J. Nutr. Biochem..

[58] Sahin K., Tuzcu M., Gencoglu H., Dogukan A., Timurkan M., Sahin N., Aslan A., Kucuk O. (2010). Epigallocatechin-3-gallate activates Nrf2/HO-1 signaling pathway in cisplatin-induced nephrotoxicity in rats. Life Sci..

[59] Romeo L., Intrieri M., D’Agata V., Mangano N.G., Oriani G., Ontario M.L., Scapagnini G. (2009). The major green tea polyphenol, (−)-epigallocatechin-3-gallate, induces heme oxygenase in rat neurons and acts as an effective neuroprotective agent against oxidative stress. Am. Coll. Nutr..

[60] Shin D.W., Kim S.N., Lee S.M., Lee W., Song M.J., Park S.M., Lee T.R., Baik J.H., Kim H.K., Hong J.H. (2009). (−)-Catechin promotes adipocyte differentiation in human bone marrow mesenchymal stem cells through PPARγ transactivation. Biochem. Pharmacol..

[61] Takahashi M., Miyashita M., Suzuki K., Bae S.R., Kim H.K., Wakisaka T., Matsui Y., Takeshita M., Yasunaga K. (2014). Acute ingestion of catechin-rich green tea improves postprandial glucose status and increases serum thioredoxin concentrations in postmenopausal women. Br. J. Nutr..

[62] Subramanian N., Venkatesh P., Ganguli S., Sinkar V.P. (1999). Role of polyphenol oxidase and peroxidase in the generation of black tea theaflavins. J. Agric. Food Chem..

[63] Stodt U.W., Blauth N., Niemann S., Stark J., Pawar V., Jayaraman S., Koek J., Engelhardt U.H. (2014). Investigation of processes in black tea manufacture through model fermentation (oxidation) experiments. J. Agric. Food Chem..

[64] Maron D.J., Lu G.P., Cai N.S., Wu Z.G., Li Y.H., Chen H., Zhu J.Q., Jin X.J., Wouters B.C., Zhao J. (2003). Cholesterol-lowering effect of a theaflavin-enriched green tea extract: A randomized controlled trial. Arch. Intern. Med..

[65] Miyata Y., Tamaru S., Tanaka T., Tamaya K., Matsui T., Nagata Y., Tanaka K. (2013). Theflavins and theasinensin A derived from fermented tea have antihyperglycemic and hypotriacylglycerolemic effects in KK-A(y) mice and Sprague-Dawley rats. J. Agric. Food Chem..

[66] Satoh T., Igarashi M., Yamada S., Takahashi N., Watanabe K. (2015). Inhibitory effect of black tea and its combination with acarbose on small intestinal α-glucosidase activity. J. Ethnopharmacol..

[67] Rodríguez-Ramiro I., Ramos S., Bravo L., Goya L., Martín M.Á. (2011). Procyanidin B2 and a cocoa polyphenolic extract inhibit acrylamide-induced apoptosis in human Caco-2 cells by preventing oxidative stress and activation of JNK pathway. J. Nutr. Biochem..

[68] Oboh G., Ademosun A.O., Ademiluyi A.O., Omojokun O.S., Nwanna E.E., Longe K.O. (2014). *In Vitro* studies on the antioxidant property and inhibition of α-amylase, α-glucosidase, and angiotensin I-converting enzyme by polyphenol-rich extracts from cocoa (theobroma cacao) bean. Pathol. Res. Int..

[69] Mellor D.D., Sathyapalan T., Kilpatrick E.S., Beckett S., Atkin S.L. (2010). High-cocoa polyphenol-rich chocolate improves HDL cholesterol in Type 2 diabetes patients. Diabet. Med..

[70] Mellor D.D., Madden L.A., Smith K.A., Kilpatrick E.S., Atkin S.L. (2013). High-polyphenol chocolate reduces endothelial dysfunction and oxidative stress during acute transient hyperglycaemia in Type 2 diabetes: A pilot randomized controlled trial. Diabet. Med..

[71] Kameya H., Ukai M. (2012). Hydroxyl Radical Scavenging Ability of Instant Coffee Evaluated by ESR Spin Trapping. J. Cook. Sci. Jpn..

[72] Predes F.S., Ruiz A.L., Carvalho J.E., Foglio M.A., Dolder H. (2011). Antioxidative and *in vitro* antiproliferative activity of *Arctium lappa* root extracts. BMC Complement. Altern. Med..

[73] Sueishi Y., Hori M., Ishikawa M., Matsu-Ura K., Kamogawa E., Honda Y., Kita M., Ohara K. (2014). Scavenging rate constants of hydrophilic antioxidants against multiple reactive oxygen species. J. Clin. Biochem. Nutr..

[74] Laranjinha J.A., Almeida L.M., Madeira V.M. (1994). Reactivity of dietary phenolic acids with peroxyl radicals: Antioxidant activity upon low density lipoprotein peroxidation. Biochem. Pharmacol..

[75] Tang Y.Z., Liu Z.Q. (2008). Chemical kinetic behavior of chlorogenic acid in protecting erythrocyte and DNA against radical-induced oxidation. J. Agric. Food Chem..

[76] Kamitani Y., Iwai K., Fukunaga T., Kimura R., Nakagiri O. (2009). *In vitro* Analysis on Inhibitory Activity of Amylolytic Enzymes in Decaffeinated Green Coffee Bean Extracts and Contributions of Chlorogenic Acids. Nippon Shokuhin Kagaku Kogaku Kaishi..

[77] Ota N., Soga S., Murase T., Shimotoyodome A., Hase T. (2010). Consumption of Coffee Polyphenols Increases Fat Utilization in Humans. J. Health Sci..

[78] Jokura H., Watanabe I., Umeda M., Hase T., Shimotoyodome A. (2015). Coffee polyphenol consumption improves postprandial hyperglycemia associated with impaired vascular endothelial function in healthy male adults. Nutr. Res..

[79] Nagao T., Ochiai R., Watanabe T., Kataoka K., Komikado M., Tokimitsu I., Tsuchida T. (2009). Visceral Fat-reducing Effect of Continuous Coffee Beverage Consumption in Obese Subjects. Jpn. Pharmacol. Ther..

[80] Wan C.W., Wong C.N., Pin W.K., Wong M.H., Kwok C.Y., Chan R.Y., Yu P.H., Chan S.W. (2013). Chlorogenic acid exhibits cholesterol lowering and fatty liver attenuating properties by up-regulating the gene expression of PPAR-α in hypercholesterolemic rats induced with a high-cholesterol diet. Phytother. Res..

[81] Li S.Y., Chang C.Q., Ma F.Y., Yu C.L. (2009). Modulating effects of chlorogenic acid on lipids and glucose metabolism and expression of hepatic peroxisome proliferator-activated receptor-alpha in golden hamsters fed on high fat diet. Biomed. Environ. Sci..

[82] Hirata A., Murakami Y., Shoji M., Kadoma Y., Fujisawa S. (2005). Kinetics of radical-scavenging activity of hesperetin and hesperidin and their inhibitory activity on COX-2 expression. Anticancer. Res..

[83] Emim J.A., Oliveira A.B., Lapa A.J. (1994). Pharmacological evaluation of the anti-inflammatory activity of a citrus bioflavonoid, hesperidin, and the isoflavonoids, duartin and claussequinone, in rats and mice. J. Pharm. Pharmacol..

[84] Akiyama S., Katsumata S., Suzuki K., Nakaya Y., Ishimi Y., Uehara M. (2009). Hypoglycemic and hypolipidemic effects of hesperidin and cyclodextrin-clathrated hesperetin in Goto-Kakizaki rats with type 2 diabetes. Biosci. Biotechnol. Biochem..

[85] Jung U.J., Lee M.K., Jeong K.S., Choi M.S. (2004). The hypoglycemic effects of hesperidin and naringin are partly mediated by hepatic glucose-regulating enzymes in C57BL/KsJ-db/db mice. J. Nutr..

[86] Kakadiya J., Mulani H., Shah N. (2010). Protective effect of hesperidin on cardiovascular complication in experimentally induced myocardial infarction in diabetes in rats. J. Basic. Clin. Pharm..

[87] Monforte M.T., Trovato A., Kirjavainen S., Forestieri A.M., Galati E.M., Lo Curto R.B. (1995). Biological effects of hesperidin, a Citrus flavonoid. (note II): Hypolipidemic activity on experimental hypercholesterolemia in rat. Farmaco.

[88] Agrawal Y.O., Sharma P.K., Shrivastava B., Ojha S., Upadhya H.M., Arya D.S., Goyal S.N. (2014). Hesperidin produces cardioprotective activity via PPAR-γ pathway in ischemic heart disease model in diabetic rats. PLoS ONE.

[89] Kadota K., Semba K., Shakudo R., Sato H., Deki Y., Shirakawa Y., Tozuka Y. (2016). Inhibition of photodegradation of highly dispersed folic acid nanoparticles by the antioxidant effect of transglycosylated rutin. J. Agric. Food Chem..

[90] Miwa Y., Mitsuzumi H., Yamada M., Arai N., Tanabe F., Okada K., Kubota M., Chaen H., Sunayama T., Kibata M. (2006). Suppression of apolipoprotein B secretion from HepG2 cells by glucosyl hesperidin. J. Nutr. Sci. Vitaminol..

[91] Miwa Y., Mitsuzumi H., Sunayama T., Yamada M., Okada K., Kubota M., Chaen H., Mishima Y., Kibata M. (2005). Glucosyl hesperidin lowers serum triglyceride level in hypertriglyceridemic subjects through the improvement of very low-density lipoprotein metabolic abnormality. J. Nutr. Sci. Vitaminol..

[92] Miwa Y., Yamada M., Sunayama T., Mitsuzumi H., Tsuzaki Y., Chaen H., Mishima Y., Kibata M. (2004). Effects of glucosyl hesperidin on serum lipids in hyperlipidemic subjects: Preferential reduction in elevated serum triglyceride level. J. Nutr. Sci. Vitaminol..

[93] Murakami A., Nakamura Y., Ohto Y., Yano M., Koshiba T., Koshimizu K., Tokuda H., Nishino H., Ohigashi H. (2000). Suppressive effects of citrus fruits on free radical generation and nobiletin, an anti-inflammatory polymethoxyflavonoid. Biofactors.

[94] Mulvihill E.E., Assini J.M., Lee J.K., Allister E.M., Sutherland B.G., Koppes J.B., Sawyez C.G., Edwards J.Y., Telford D.E., Charbonneau A. (2011). Nobiletin attenuates VLDL overproduction, dyslipidemia, and atherosclerosis in mice with diet-induced insulin resistance. Diabetes.

[95] Nii Y., Okahisa N., Takata J., Mino Y., Shikishima Y. (2014). Sudachitin Contents and Antioxidative Activities of Sudachi Peel Extracts. Rep. Tokushima Prefect. Ind. Technol. Cent..

[96] Tsutsumi R., Yoshida T., Nii Y., Okahisa N., Iwata S., Tsukayama M., Hashimoto R., Taniguchi Y., Sakaue H., Hosaka T. (2014). Sudachitin, a polymethoxylated flavone, improves glucose and lipid metabolism by increasing mitochondrial biogenesis in skeletal muscle. Nutr. Metab..

[97] Miyake Y., Mochizuki M., Okada M., Hiramitsu M., Morimitsu Y., Osawa T. (2007). Isolation of antioxidative phenolic glucosides from lemon juice and their suppressive effect on the expression of blood adhesion molecules. Biosci. Biotechnol. Biochem..

[98] Minato K., Miyake Y., Fukumoto S., Yamamoto K., Kato Y., Shimomura Y., Osawa T. (2003). Lemon flavonoid, eriocitrin, suppresses exercise-induced oxidative damage in rat liver. Life Sci..

[99] Fritz H., Seely D., Flower G., Skidmore B., Fernandes R., Vadeboncoeur S., Kennedy D., Cooley K., Wong R., Sagar S. (2013). Soy, red clover, and isoflavones and breast cancer: A systematic review. PLoS ONE.

[100] Han R.M., Tian Y.X., Liu Y., Chen C.H., Ai X.C., Zhang J.P., Skibsted L.H. (2009). Comparison of flavonoids and isoflavonoids as antioxidants. J. Agric. Food Chem..

[101] Zhang T., Wang F., Xu H.X., Yi L., Qin Y., Chang H., Mi M.T., Zhang Q.Y. (2013). Activation of nuclear factor erythroid 2-related factor 2 and PPARγ plays a role in the genistein-mediated attenuation of oxidative stress-induced endothelial cell injury. Br. J. Nutr..

[102] Takashima M., Nara K., Niki E., Yoshida Y., Hagihara Y., Stowe M., Horie M. (2013). Evaluation of biological activities of a groundnut (Apios americana Medik) extract containing a novel isoflavone. Food Chem..

[103] Ko K.P., Kim C.S., Ahn Y., Park S.J., Kim Y.J., Park J.K., Lim Y.K., Yoo K.Y., Kim S.S. (2015). Plasma isoflavone concentration is associated with decreased risk of type 2 diabetes in Korean women but not men: Results from the Korean Genome and Epidemiology Study. Diabetologia.

